# Allergic Cheilitis to Cane Reed in a Teenage Oboe Player

**DOI:** 10.1111/cod.70217

**Published:** 2026-07-10

**Authors:** Sarah Milesi, Laura Lemaire, Françoise Giordano

**Affiliations:** ^1^ Service de Dermatologie CHU Toulouse Toulouse France

**Keywords:** cane reed, case report, cheilitis, contact cheilitis, lip diseases, oboe

Allergic contact dermatitis associated with musical instrument playing is an underestimated occupational condition, despite musicians' exposure to multiple potential allergens.

## 1 Case Report

We report the case of a 13‐year‐old boy with a history of atopic dermatitis during early childhood. He had been playing oboe for 7 years at a high level and was referred to us for scaling cheilitis, sometimes associated with pustular lesions (Figure 1). The lesions had appeared 9 months earlier, mainly involved the center of the lower lip (corresponding to the contact area with the cane reed), and interfered with his ability to play. He reported applying only Dexpanthenol ointment topically and noticed improvement of the lesions when he didn't play oboe for several days. Patch testing was performed with the European baseline, cosmetic, and cheilitis series using IQ Ultra chambers. His toothpaste, Dexpanthenol ointment, and varnished and unvarnished patient's oboe reed fragments “as is” in petrolatum were also tested (Figure 2a). Readings on days 2 and 3 revealed a strong positive reaction (2+) exclusively for the unvarnished reed (Figure 2b), confirming allergic contact dermatitis to the cane rather than to the varnish.

**FIGURE 1:**
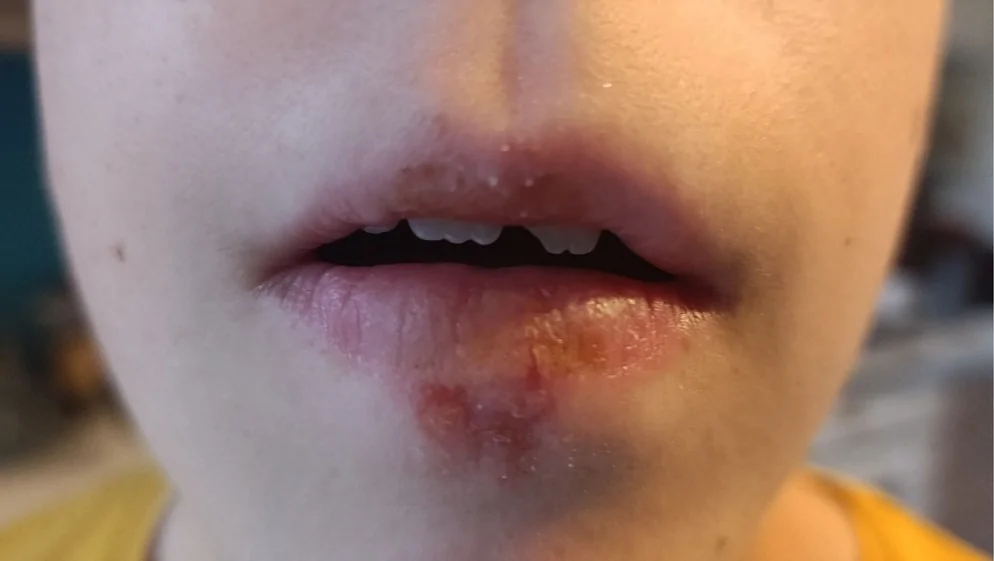
Crusty, cracked cheilitis and eczematous lesions corresponding to the areas where the reed was applied.

**FIGURE 2:**
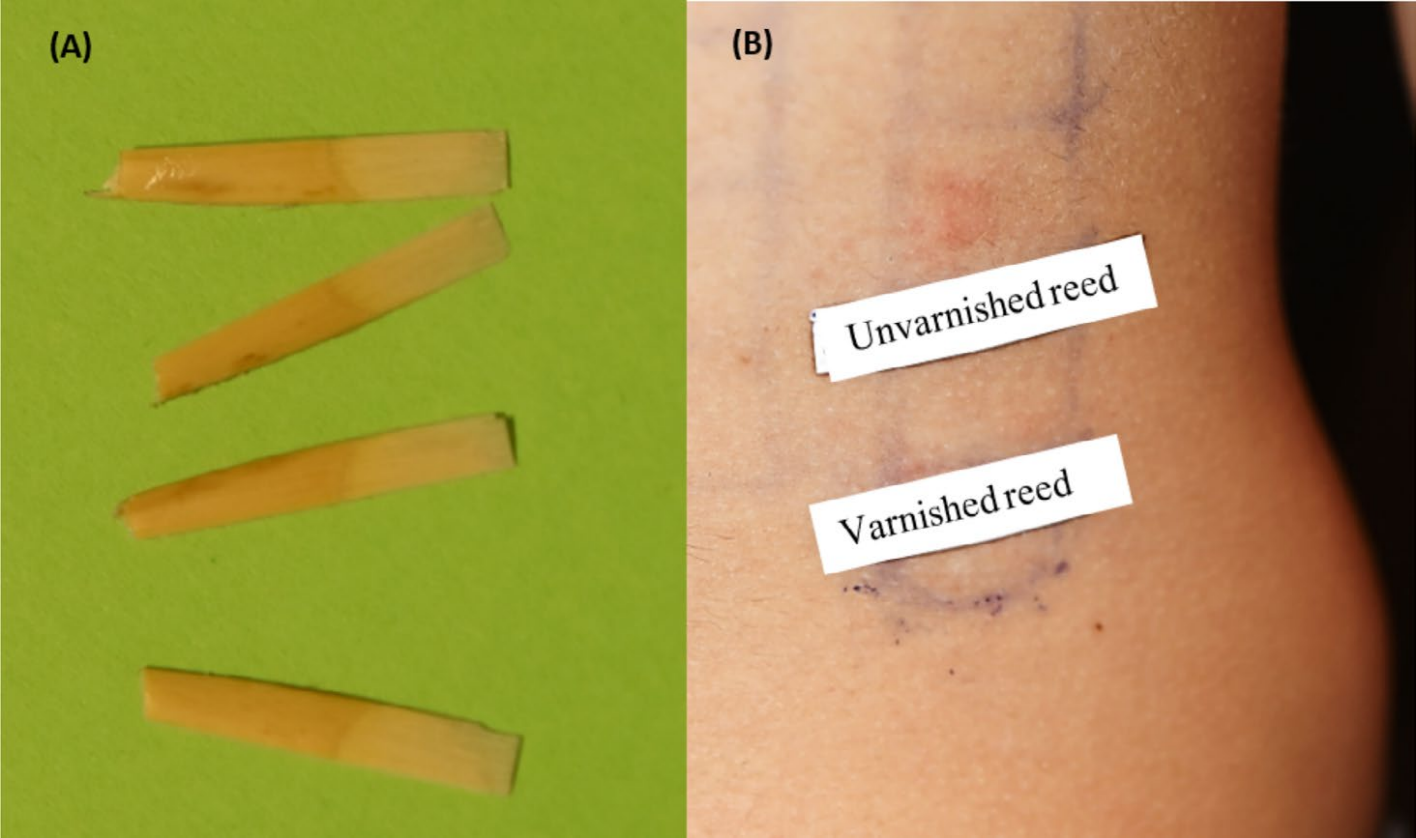
(A) Cane reed. The lightest area corresponds to the part without varnish. (B) Positive patch test for unvarnished reed (top).

## 2 Discussion

Wind instrument players are particularly affected by contact cheilitis due to prolonged contact between the instrument, perioral skin, and oral mucosa [1]. Although this condition remains uncommon, only a few cases of contact dermatitis related to wind instrument reeds have been reported, mainly among clarinettists and saxophonists. To our knowledge, we report the first case of contact cheilitis to cane reed occurring in an oboist.

Oboe reeds are traditionally handmade from natural cane ( *Arundo donax* ), also known as Provence cane. To improve durability, reeds may be treated with varnishes or coatings.

In 1986, Friedman and Connolly [2] described “clarinettist's cheilitis,” a median cheilitis of the lower lip associated with *A.d*. reed. As patch testing was negative, the authors attributed the condition to local irritants. Later reports [3, 4, 5] documented delayed‐type contact allergy to *A.d*. reeds, with positive patch tests. In contrast, Ruiz et al. [6] described an immediate‐type reaction resembling contact urticaria in an atopic clarinettist exposed to an *A.d*. reed with positive skin prick test but negative patch tests.

However, the vibrating surface in contact with lips must remain uncoated to allow moisture absorption and proper acoustic performance. Our patient was advised to consider plastic or resin reeds as an alternative but these are often considered acoustically inferior and may be unsuitable for advanced or professional musicians.

## Author Contributions

**Sarah Milesi:** conceptualization, investigation, writing – original draft, funding acquisition, methodology, writing – review and editing. **Françoise Giordano:** conceptualization, investigation, validation, supervision. **Laura Lemaire:** conceptualization, investigation.

## Consent

Informed consent was obtained from the patient used in this study.

## Conflicts of Interest

The authors declare no conflicts of interest.

## Data Availability

The data that support the findings of this study are available from the corresponding author upon reasonable request.
